# Kaempferol inhibits *Chlamydia psittaci* proliferation by blocking lipid transport and RB-EB differentiation

**DOI:** 10.3389/fmicb.2026.1783916

**Published:** 2026-03-13

**Authors:** Yinhui Lin, Yufei Jiang, Shan Zhang, Ziyuan Zhang, Shaoya Liu, Jinru Lin, Xiaoxiao Chen, Yuchen Zhang, Yonghui Yu, Wenbo Wei, Xuan OuYang, Xinan Huang, Yajun Song, Jun Jiao

**Affiliations:** 1 Artemisinin Research Center, Guangzhou University of Chinese Medicine, Guangzhou, China; 2 State Key Laboratory of Pathogen and Biosecurity, Academy of Military Medical Sciences, Beijing, China

**Keywords:** *Chlamydia psittaci*, developmental cycle, kaempferol, lipid droplets, RB-EB differentiation

## Abstract

*Chlamydia psittaci* is an obligate intracellular pathogen that poses a significant threat to both human and animal health. The current therapeutic strategies are limited by the emergence of potential drug resistance, underscoring the urgent need for novel anti-chlamydial agents. In this study, we evaluated the anti-chlamydial activity of kaempferol, a natural occurring flavonoid. Potential targets of kaempferol and *C. psittaci* infection-related targets were identified through *in silico* screening. In cellular assays using both HeLa and THP-1 cells, kaempferol treatment significantly inhibits the intracellular growth of *C. psittaci* in a dose-dependent manner, while showing no host cell cytotoxicity at effective concentrations. Based on prior in silico virtual screening and our group’s systematic evaluation of the anti-chlamydial activity of flavonoids, kaempferol (40 μM) was selected as the target compound for this study due to its potent antimicrobial effects and its potential to modulate key host–pathogen interaction pathways. Mechanistic investigations revealed that kaempferol interferes with the trafficking of host-derived lipid to the bacterial inclusion, thereby depriving the pathogen of essential nutrients. Moreover, kaempferol treatment severely disrupted the normal differentiation of reticulate bodies (RBs) into infectious elementary bodies (EBs), resulting in an aberrant developmental cycle and a reduction in the production of new infectious progeny. This study demonstrates that kaempferol exerts anti-chlamydial activity by targeting two key processes of *C. psittaci* infection: lipid trafficking to inclusions and RB-EB conversion. These results highlight kaempferol as a promising lead compound for developing new therapeutic approaches against *C. psittaci* infections.

## 1 Introduction

Psittacosis is a significant zoonotic disease, caused by an obligate intracellular Gram-negative bacterium *Chlamydia psittaci* (Knittler and Sachse, 2015). The clinical manifestations of psittacosis range from mild, non-specific manifestations to systemic illness, often complicated by severe pneumonia (Kong et al., 2021). Like other *Chlamydia* species, *C. psittaci* exhibits a biphasic developmental cycle lasting 36 to 72 h (Knittler and Sachse, 2015). Infection begins when the infectious elementary body (EB; ~0.2 μm in diameter) adheres to and enters the host cell, becoming enclosed within a membrane-bound compartment termed an inclusion (Escalante-Ochoa et al., 1998; Wang et al., 2024). Inside the inclusion, EBs differentiate into reticulate bodies (RBs; ~0.8 μm in diameter), which are metabolically active but non-infectious forms (Panzetta et al., 2018). The RBs then undergo proliferation by binary fission, utilizing host-derived ATP and metabolites. Following 8 to 12 division cycles, RBs re-differentiate into new EBs, which are released from the host cell via lysis or extrusion (Bastidas et al., 2013; Knittler et al., 2014). These mature EBs can subsequently infect neighboring cells, thereby completing the developmental cycle.

The obligate intracellular lifestyle of *C. psittaci* renders it heavily dependent on host-derived nutrients. A key aspect of this dependency is the pathogen’s ability to hijack host cellular resources, such as lipids, and divert them to the inclusion where the bacteria replicate (Kellermann et al., 2021; Husler et al., 2023). Moreover, successful infection requires the precise differentiation of RBs back into infectious EBs. This RB-to-EB transition is tightly regulated, and its disruption halts the production of new infectious progeny, preventing pathogen dissemination. Therefore, therapeutic strategies targeting either this developmental switch or the pathogen’s nutrient acquisition pathways hold significant promise for combating chlamydial infections.

This review systematically summarizes recent advances in the molecular epidemiology, host range, and emerging risk of human-to-human transmission of *Chlamydia psittaci* (Wang et al., 2024). Currently, clinical treatment of *C. psittaci* infections relies primarily on antibiotics such as tetracyclines and macrolides (Centers for Disease Control and Prevention, 2000; Wang et al., 2024; Yang et al., 2025). However, increasing reports of antibiotic-resistant *C. psittaci* strains and side effects of long-term antibiotic use have driven the search for safer and more effective alternatives (Benamri et al., 2021; Alsubaiyel and Bukhari, 2024; Qin et al., 2024). In recent years, traditional Chinese medicine (TCM) has shown distinctive advantages in anti-infective therapy, offering novel strategies against drug-resistant and intracellular pathogens (Mu et al., 2025; SeyedAlinaghi et al., 2025). The anti-infective efficacy of TCM extends beyond the direct inhibition or killing of pathogens; it also enhances host tolerance to infection through mechanisms such as clearing heat and removing toxins, reinforcing healthy qi, and protecting organ function. By constructing a mathematical model of host–pathogen interaction, previous study demonstrates that eliminating the pathogen alone does not necessarily lead to host recovery. Instead, host-directed strategies that “fortify vital energy and balance immunity” are critical for improving infection outcomes (Sun, 2023). In clinical practice, TCM management of psittacosis emphasizes clearing heat-toxins, diffusing the lung, and resolving phlegm. Various prescriptions are derived from classical formulas, national guideline protocols, and clinically experienced formulations. Core herbs commonly used in such formulations include *Ephedra sinica* (Má Huáng), *Prunus armeniaca* (Xìng Rén), *Scutellaria baicalensis* (Huáng Qín), and *Forsythia suspensa* (Lián Qiào) (Li et al., 2018; Zheng et al., 2022).

Kaempferol (Kae) is a natural flavonoid abundantly present in the rhizome of *Alpinia galanga* (a member of Zingiberaceae) as well as in various vegetables and fruits. It exhibits a broad spectrum of pharmacological activities, including antitumor (Imran et al., 2019), anti-inflammatory (Alam et al., 2020), antioxidant (Hussain et al., 2024), and cardioprotective effects. Additionally, kaempferol and its derivatives have demonstrated antibacterial, antifungal, and antiparasitic properties (Periferakis et al., 2022). Flavonoid compounds such as quercetin and kaempferol have been explored for their potential application value as non-antibiotic strategies in inhibiting *Chlamydia* infection (Hou et al., 2022). Despite this broad-spectrum potential, its specific efficacy and mechanism of action against *C. psittaci* remain largely unexplored. In this study, we combined network pharmacology with experimental validation to identify kaempferol, a bioactive constituent of TCM, as a potential anti-*C. psittaci* agent, and to preliminarily clarify its underlying molecular mechanisms.

## 2 Materials and methods

### 2.1 Bacterial strains and cell culture

The human cervical cancer cell line (HeLa) and human monocyte leukemia cell line (THP-1) were obtained from the American Type Culture Collection (ATCC). HeLa cells were cultured in Dulbecco’s Modified Eagle’s Medium (DMEM) containing 10% fetal bovine serum (FBS) at 37 °C in a 5% CO_2_ incubator. THP-1 cells were cultured in RPMI 1640 medium containing 10% FBS at 37 °C in a 5% CO_2_ incubator. The *C. psittaci* 6 BC strain and GFP-expressing *C. psittaci* strain (Li et al., 2025) were propagated in HeLa cells at 37 °C in a 5% CO_2_ incubator.

### 2.2 Reagents

Kaempferol (3,4′,5,7-tetrahydroxyflavone, HPLC purity ≥97%) was acquired from ALADDIN (Shanghai, China). Quercetin (3,3′,4′,5,7-Pentahydroxyflavone, HPLC purity ≥97%) and Luteolin (3′,4′,5,7-Tetrahydroxyflavone, HPLC purity ≥98%) were acquired from Macklin (Shanghai, China). Doxycycline was acquired from MCE (New Jersey, NJ, USA). BODIPY 493/503 and Lipofectamine 8000™ transfection reagent were purchased from Beyotime (Shanghai, China). ProLong Glass Antifade Mountant with NucBlue was obtained from Invitrogen (Carlsbad, CA, USA). The plasmid pCMV-CERT1 (Ceramide Transfer Protein, human)-EYFP-Neo was procured from Solarbio (Beijing, China). The Cell Counting Kit-8 (CCK-8) was obtained from DUOMI (Beijing, China). TransScript^®^ One-Step gDNA Removal and cDNA Synthesis SuperMix was sourced from TransGen Biotech (Beijing, China). The Universal Genomic DNA Kit and RNAPure Bacteria Kit (DNase I) were provided by CWBIO (Beijing, China). PowerUP™ SYBR™ Green Master Mix and TaqMan™ Universal Master Mix II were purchased from Applied Biosystems (Foster City, CA, USA).

### 2.3 Bioinformatics analysis

Active components of *Scutellaria baicalensis*, *Forsythia suspensa*, *Prunus armeniaca*, and *Ephedra sinica* were retrieved from the TCMSP database.^1^ Potential active molecules were screened using oral bioavailability (OB) ≥ 30% and drug-likeness (DL) ≥ 0.18 as criteria. Targets of these components were predicted using the SWISSTargetPrediction platform.^2^ Disease targets associated with “Chlamydia pneumonia, Psittacosis, ornithosis” were collected from the Genecards^3^ and OMIM^4^ databases, integrated, and deduplicated (Beeckman and Vanrompay, 2009; Sreiri et al., 2024). The Venny 2.1.0 tool^5^ was used to identify common targets by intersecting the potential targets of the active components with the disease-related targets.

Subsequently, the common targets were imported into the STRING 11.5^6^ database to construct a protein–protein interaction (PPI) network, with the species set to “*Homo sapiens*” and a confidence score threshold ≥0.7. Disconnected nodes were hidden to highlight key interactions. The PPI network was then visualized and topologically analyzed using Cytoscape 3.10.0 to identify core targets. The cytoHubba plugin in Cytoscape was utilized for degree scoring, and targets were ranked based on their degree values.

Finally, the DAVID 6.8 platform was used to perform Gene Ontology (GO) functional enrichment analysis and Kyoto Encyclopedia of Genes and Genomes (KEGG) pathway analysis on the intersecting core targets, with a significance threshold of *p* < 0.05.

### 2.4 Cell viability assay

HeLa and THP-1 cells were seeded in 96-well plates at 5 × 10^4^ cells/mL, with THP-1 cells primed with 200 nM PMA to induce adhesion. After 24 h of culture at 37 °C with 5% CO₂, cells were treated with quercetin (16, 32, or 64 μM), kaempferol (20, 40, or 60 μM), or luteolin (10, 20, or 40 μM), using DMSO treated wells as controls. Following 24 h or 48 h of incubation, a CCK-8 working solution (9:1 basal medium to CCK-8 reagent) was added to each well. After 30 min of incubation, absorbance was measured at 450 nm using a microplate reader.

### 2.5 Intracellular growth curve analysis

HeLa and THP-1 cells were seeded in 12-well plates at 1 × 10^6^ cells/mL, with THP-1 cells primed with 200 nM PMA to induce adhesion. Following the attachment, cells were infected with *C. psittaci* at an MOI (multiplicity of infection) of 1, centrifuged at 900 × *g* for 10 min, and incubated at 37 °C for 3 h to facilitate bacterial entry. Infected cells were then treated with kaempferol (20, 40, or 60 μM), with DMSO treated as control. Cells were harvested at 36 h and 48 h post-infection, and genomic DNA was extracted using the Universal Genomic DNA Kit. Bacterial loads were quantified by TaqMan probe-based quantitive PCR (qPCR) (Table 1) under the following cycling conditions: 50 °C for 2 min; 95 °C for 10 min; 40 cycles of 95 °C for 15 s and 60 °C for 1 min.

**Table 1:** Primers of quantitative PCR.

No.	Primers	Sequence (5′ → 3′)
1	CPs-Forward	CCCACATAGTGCCATCGAT
	CPs-Reverse	GGTTCCGCTCTCTCCTTACAAG
	CPs-Probe	5′6-FAM-TGCCTGTAGGGAACCCAGCTGAAC-3’-BHQ1
2	gyrb-Forward	AGGCAGCAAAGAAGGCTAGG
	gyrb-Reverse	CTTAGCAGATCCTCCAGCGG
	euo-Forward	ACAGAGAAGATGCGCAGCAA
	euo-Reverse	GATCGATTTCCCACCGGGTT
	omcB-Forward	ACTCAACAGCTTCCTTGCGA
	omcB-Reverse	ATACAGTAGCCGCGGTGAAG
	hctB-Forward	TGCCACAAGCATCACAGACA
	hctB-Reverse	ATTGTTGACGCCAGCCATGA
	incC-Forward	TCGCTACCTCCATTAAGTGC
	incC-Reverse	ATTGTTCCAGATGCTGTGCC

1, for qPCR; 2, for RT-qPCR.

### 2.6 Kaempferol and doxycycline intervention experiment

HeLa cells were seeded in 12-well plates at a density of 1 × 10^6^ cells/mL. Following adhesion, cells were infected with *C. psittaci* at a multiplicity of infection (MOI) of 1, centrifuged at 900 × g for 10 min, and incubated at 37 °C for 3 h to facilitate bacterial entry. Four experimental groups were established: DMSO control, kaempferol (40 μM), doxycycline (0.02 μM), and a combination of kaempferol and doxycycline (40 μM + 0.02 μM). Cells were harvested at 36 and 48 h post-infection, and genomic DNA was extracted using the Universal Genomic DNA Kit. Bacterial loads were quantified by TaqMan probe-based quantitative PCR (qPCR) (Table 1) under the following cycling conditions: 50 °C for 2 min; 95 °C for 10 min; 40 cycles of 95 °C for 15 s and 60 °C for 1 min.

### 2.7 Lipid transport assay

HeLa cells were seeded on glass coverslips in 24-well plates at 1 × 10^5^ cells/mL. After the adherence, cells were infected with *C. psittaci* at an MOI of 1, centrifuged at 900 × *g* for 10 min, and incubated at 37 °C for 3 h to facilitate infection. Cells were then treated with 40 μM kaempferol, with DMSO treated as control. At 36 h post-infection, cells were washed three times with PBS to remove debris, fixed with 4% paraformaldehyde for 10 min at room temperature, and washed again with PBS. Lipid droplets were stained using BODIPY 493/503 for 10 min, followed by PBS washes. Coverslips were mounted with Hoechst-containing medium and imaged by confocal laser scanning microscopy (Olympus, FV3000).

### 2.8 Co-localization analysis

HeLa cells were seeded on glass coverslips in 24-well plates at 1 × 10^5^ cells/mL and transfected with CERT-EYFP plasmid using Lipofectamine™ 8000 for 24 h. Cells were then infected with *C. psittaci* at an MOI of 1 and treated with 40 μM kaempferol, with DMSO treated as control. At 36 h post-infection, samples were washed three times with PBS to remove cellular debris, fixed with 4% paraformaldehyde for 10 min at room temperature, and permeabilized with 0.1% Triton X-100 for 15 min. After additional PBS washes, coverslips were mounted with Hoechst-containing medium and visualized by confocal laser scanning microscopy (Olympus, FV3000).

### 2.9 Transmission electron microscopy (TEM)

HeLa cells were seeded on glass coverslips in 6-well plates at 1 × 10^6^ cells/mL and infected with *C. psittaci* at an MOI of 1. The experimental group was treated with 40 μM kaempferol, with DMSO treated as control. At 36 h post-infection, samples were collected and fixed with pre-chilled 2.5% glutaraldehyde at 4 °C for 24 h. After fixation in electron microscopy preservation solution, developmental transitions of *C. psittaci* were examined by transmission electron microscopy (Zhongke Baice Co., Ltd.).

### 2.10 Reverse transcription quantitative PCR (RT-qPCR)

HeLa cells were seeded on glass coverslips in 12-well plates at 1 × 10^6^ cells/mL and infected with *C. psittaci* at an MOI of 1. Cells were treated with 40 μM kaempferol, with DMSO treated as control. Samples were collected at 12 h, 24 h, and 36 h post-infection. Total chlamydial RNA was extracted using the RNAPure Bacteria Kit (DNase I) and reverse-transcribed into cDNA with SuperMix. Transcript levels of *euo*, *hctB*, *incC*, and *omcB* were quantified by SYBR Green-based RT-qPCR (Table 1). Relative expression was calculated using the 2^−ΔΔCt^ method, with *gyrB* (DNA gyrase subunit B) as the endogenous control.

### 2.11 Statistical analysis

Statistical analysis was conducted using GraphPad Prism 8.0 software. All data of the experimental groups were obtained from three independent experiments, and results are presented as means ± standard deviation. Student’s t-test or one-way analysis of variance (ANOVA) generated a minimum threshold of significance of *p* < 0.05.

## 3 Results

### 3.1 Screening of bioactive components and common targets

Active components of *Ephedra sinica*, *Prunus armeniaca*, *Scutellaria baicalensis*, and *Forsythia suspensa* were retrieved from relevant databases, yielding a total of 70 botanical active ingredients after removing duplicates (Supplementary Table 1). Topological analysis using Cytoscape 3.7.2 identified quercetin, kaempferol, luteolin, wogonin, and naringenin as the top five key active components based on degree values (Table 2). Quercetin, kaempferol, and luteolin, which exhibited the three highest degree values, were selected for subsequent experiments.

**Table 2:** Top five active ingredients in traditional Chinese medicine.

No	Ingredient	Degree
1	Quercetin	120
2	Kaempferol	47
3	Luteolin	47
4	Wogonin	35
5	naringenin	30

A total of 216 targets corresponding to 70 active components from TCM were intersected with 529 disease-related targets obtained from disease databases, revealing 67 shared targets (Supplementary Figure S1A). The PPI network was constructed, and isolated nodes were hidden to emphasize key interactions. Using the cytoHubba plugin in Cytoscape, the top eight hub targets identified were TP53, TNF, IL6, 1 L-1β, AKT1, CXCL8, IFNG, and CCL2. Node color intensity reflected degree values, with darker shades indicating stronger network connectivity and greater functional significance (Supplementary Figure 1B).

**Figure 1:**
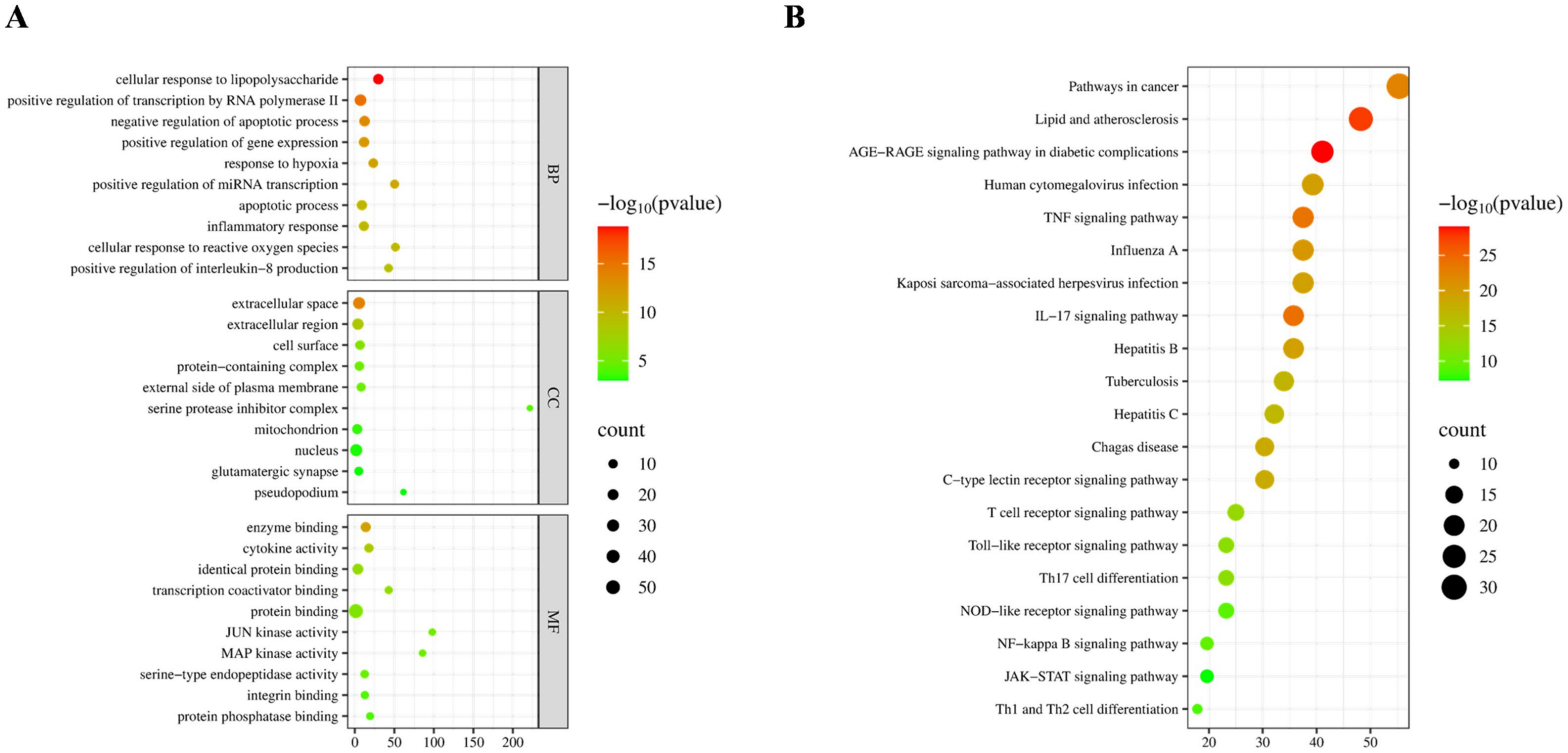
Screening of bioactive components and common targets. **(A)** Gene ontology (GO) functional enrichment analysis of common targets shared between drug components and psittacosis. **(B)** Kyoto encyclopedia of genes and genomes (KEGG) pathway enrichment analysis of the common targets.

GO enrichment analysis of the common targets via DAVID revealed significant enrichment in biological processes such as cellular response to lipopolysaccharide and negative regulation of apoptosis (Figure 1A). KEGG pathway analysis indicated significant associations with pathways including Lipid and Atherosclerosis, and AGE*-*RAGE signaling pathway in diabetic complications, with notable enrichment in lipid-related pathways (Figure 1B).

These findings suggested that flavonoids such as kaempferol, quercetin, and luteolin may inhibit intracellular proliferation of *C. psittaci* by interfering with host cell lipid metabolism and transport, thereby disrupting the lipid microenvironment essential for its intracellular survival.

### 3.2 Evaluation of cellular safety and anti-*C. psittaci* activity of kaempferol

**Figure 2:**
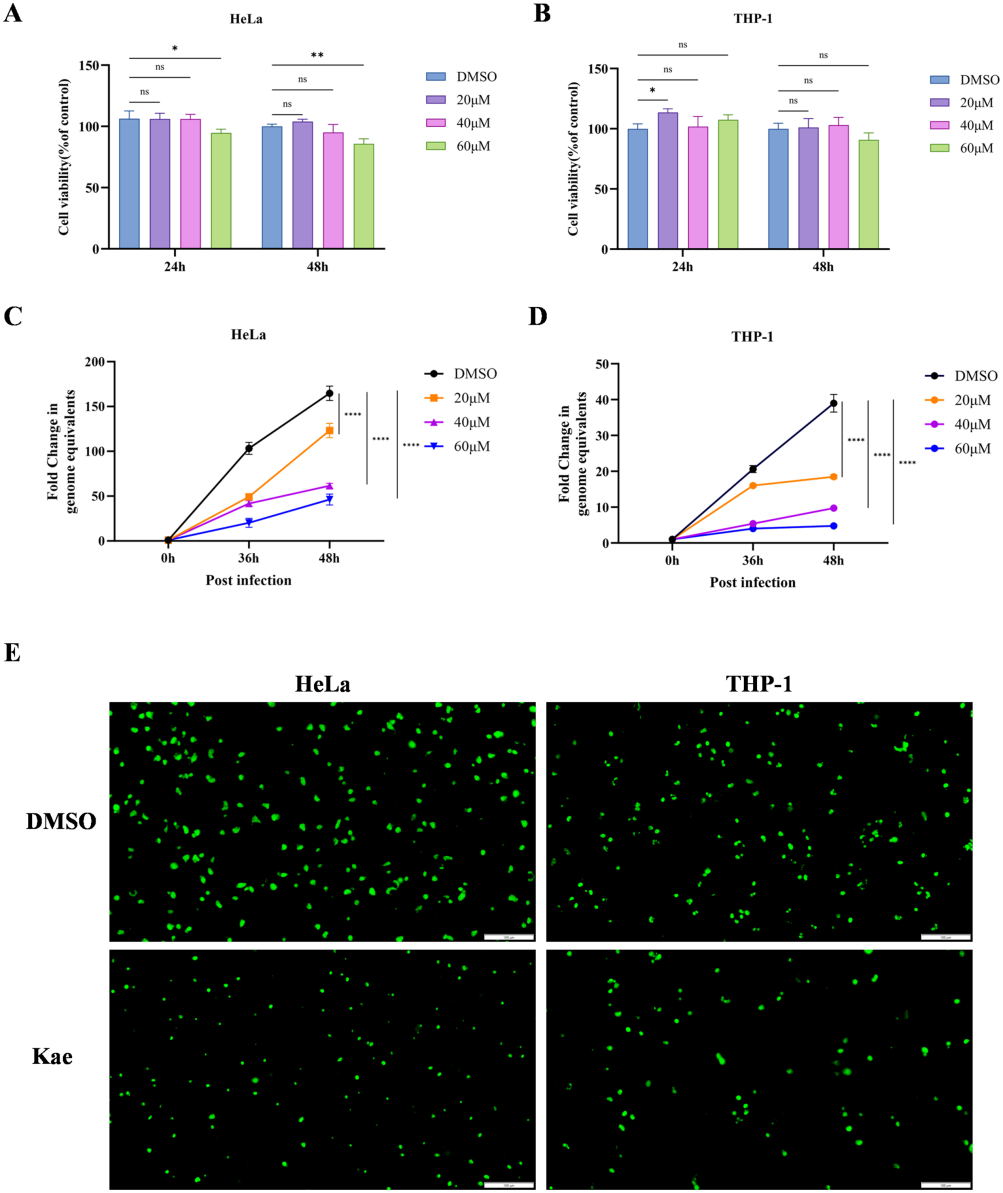
Cellular safety and anti-*C. psittaci* efficacy of kaempferol. **(A, B)** HeLa and THP-1 cells were treated with varying concentrations of kaempferol (20, 40, and 60 μM) or DMSO (control) for 24 and/or 48 h, and cell viability was assessed using the CCK-8 assay. **(C, D)** HeLa cells were infected with *C. psittaci* at an MOI of 1 for 3 h and then treated with 40 μM kaempferol or DMSO (control). The intracellular bacterial yield was assessed by qPCR at the indicated time points (36 h and 48 h). **(E)** HeLa and THP-1 cells were infected with *C. psittaci* at an MOI of 1 for 3 h, respectively, and then treated with 40 μM kaempferol or DMSO (control). The bacterial growth was visualized by fluorescence microscopy at 48 h post-infection. Scale bar = 100 μm. *p < 0.05, **p < 0.01, ***p < 0.001 vs. control group.

The effects of quercetin, kaempferol, and luteolin on HeLa and THP-1 cell viability were assessed using a CCK-8 assay. Kaempferol at the tested concentrations (20 μM, 40 μM) caused no significant adverse effects on the viability of either cell type compared with the control (Figures 2A,B). In contrast, both quercetin and luteolin exhibited concentration-dependent cytotoxicity, significantly reducing HeLa and THP-1 cell viability as concentrations increased (Supplementary Figures S2A,B).

To evaluate antibacterial activity, HeLa and THP-1 cells were infected with *C. psittaci* and treated with kaempferol. Intracellular bacterial load was measured by qPCR at various time points. Kaempferol treatment led to a significant, dose-dependent reduction in pathogen load compared with the control (Figures 2C,D). A concentration of 40 μM kaempferol markedly suppressed intracellular bacterial proliferation without evident cytotoxicity.

To visualize this effect more clearly, HeLa and THP-1 cells were infected with a GFP-expressing *C. psittaci* strain (Li et al., 2025). Intense GFP fluorescence was observed in both HeLa and THP-1 cells without kaempferol, whereas fluorescence intensity was markedly weaker in cells treated with 40 μM kaempferol, indicating inhibition of intracellular replication of *C. psittaci* (Figure 2E).

Collectively, these results demonstrate that kaempferol significantly inhibits intracellular growth of *C. psittaci* in a dose-dependent manner without host cell cytotoxicity at effective concentrations.

### 3.3 Kaempferol simultaneously disrupts the chlamydial recruitment of lipid droplets and host CERT

**Figure 3:**
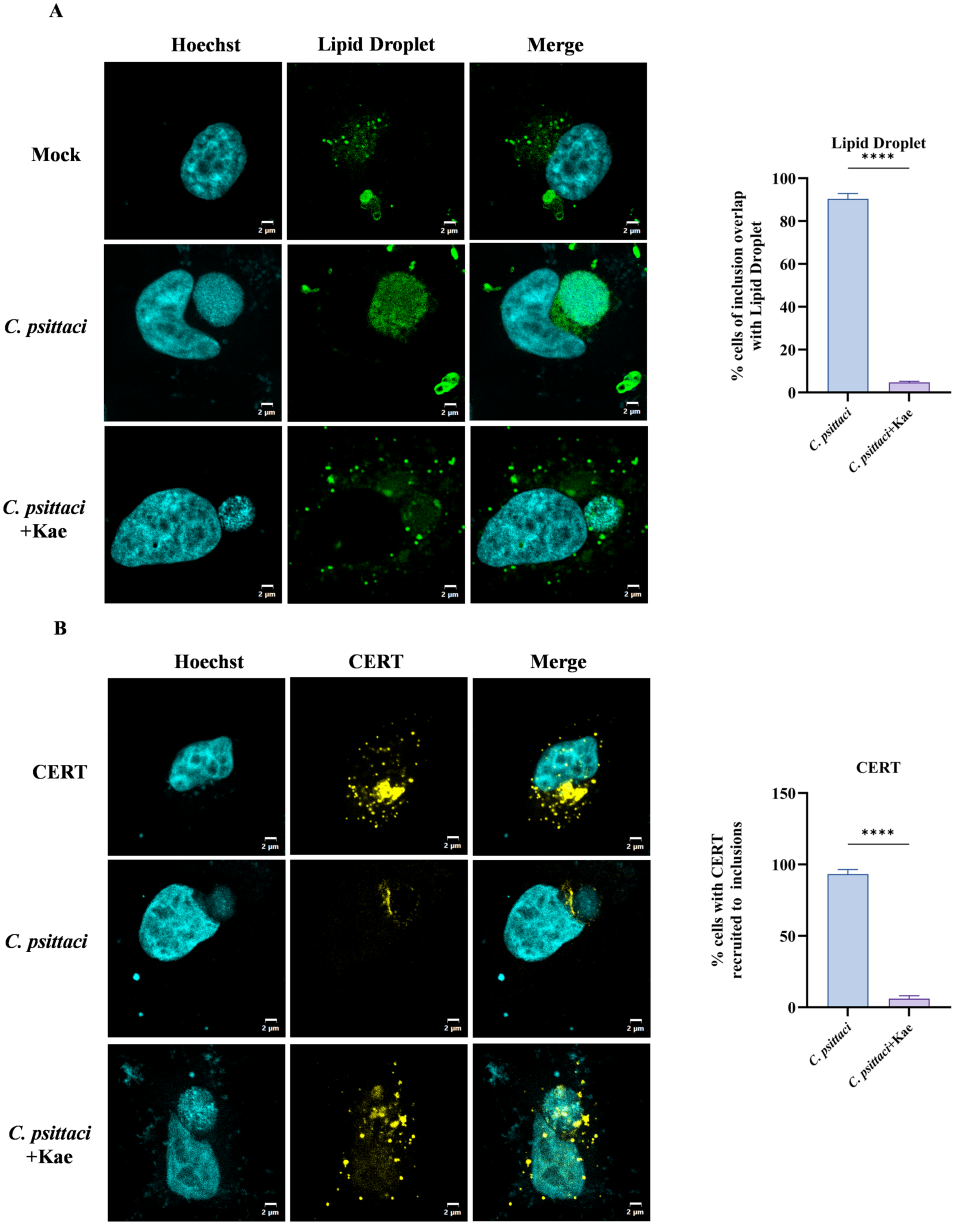
Effects of kaempferol on chlamydial recruitment of lipid droplets and host CERT. **(A)** HeLa cells were infected with *C. psittaci* at an MOI of 1 for 3 h and then treated with 40 μM kaempferol or DMSO (control). After 36 h of incubation, the cells were fixed with 4% paraformaldehyde and stained with BODIPY 493/503 to visualize lipid droplets (green). Host nuclei and bacterial DNA were counterstained with Hoechst (blue). The accumulation of lipid droplets around inclusions was observed and quantified. The experiment was repeated three times with 30 cells counted per group in each repeat; scale bar = 2 μm. **(B)** Cells transfected with CERT-EYFP (yellow) for 24 h were infected with *C. psittaci* at an MOI of 1 and then treated with 40 μM kaempferol or DMSO (control). After 36 h of incubation, samples were processed and imaged by confocal microscopy. A quantitative analysis of the co-localization is shown. The experiment was repeated three times with 30 cells counted per group in each repeat. Scale bar = 2 μm. *p < 0.05, **p < 0.01, ***p < 0.001 vs. control group.

Network pharmacology analysis suggested that kaempferol might inhibit the intracellular proliferation of *C. psittaci* by interfering with host lipid metabolism and trafficking (Figure 1). Previous studies have established that chlamydial growth depends on the transport of host lipids to the inclusion (Hackstadt et al., 1995; Elwell and Engel, 2012; Gurumurthy et al., 2014; Moldovan et al., 2025), indicating that kaempferol might exert its anti-infective effects by modulating this process. To test this hypothesis, HeLa cells infected with *C. psittaci* were treated with 40 μM kaempferol. Lipid droplets in host cells were stained with BODIPY 493/503 and visualized using confocal microscopy to evaluate their colocalization with the bacterial inclusion. In uninfected cells, lipid droplets appeared as punctate structures distributed throughout the cytoplasm (Saka et al., 2015; Monteiro-Bras et al., 2019); a similar distribution was observed in *C. psittaci*-infected cells following kaempferol treatment. In contrast, infected cells without kaempferol treatment exhibited pronounced accumulation of lipid droplets within the lumen of the chlamydial inclusion (Figure 3A), consistent with previous study (Cocchiaro et al., 2008). These findings suggest that kaempferol inhibits the recruitment of lipid droplets to the inclusion during *C. psittaci* infection.

To survive and replicate, *Chlamydia* acquires essential lipids such as sphingomyelin (SM) from the host cell during infection. The major pathway for SM biosynthesis involves transport of ceramide from the ER to the trans-Golgi by the cytosolic lipid transfer protein CERT (Hanada et al., 2003; Kawano et al., 2006). CERT-dependent trafficking of SM to the chlamydial inclusion-mediated through recruitment to the inclusion and interaction with the inclusion membrane protein IncD—has been previously demonstrated (Derre et al., 2011; Koch-Edelmann et al., 2017; Banhart et al., 2019). We therefore asked whether kaempferol affects CERT recruitment. HeLa cells expressing CERT-EYFP were infected with *C. psittaci* and treated with 40 μM kaempferol. Confocal microscopy showed that in uninfected cells and kaempferol-treated infected cells, CERT-EYFP exhibited diffuse cytoplasmic localization. In untreated infected cells, CERT-EYFP was prominently recruited to the inclusion (Figure 3B), consistent with earlier reports (Derre et al., 2011; Elwell et al., 2011). These results indicate that kaempferol disrupts the infection-induced recruitment of CERT.

Collectively, these findings demonstrate that Kaempferol simultaneously disrupts the chlamydial recruitment of lipid droplets and host CERT. This dual inhibition blocks the acquisition of key lipids.

### 3.4 Kaempferol inhibited the RB-to-EB differentiation of *C. psittaci*

**Figure 4:**
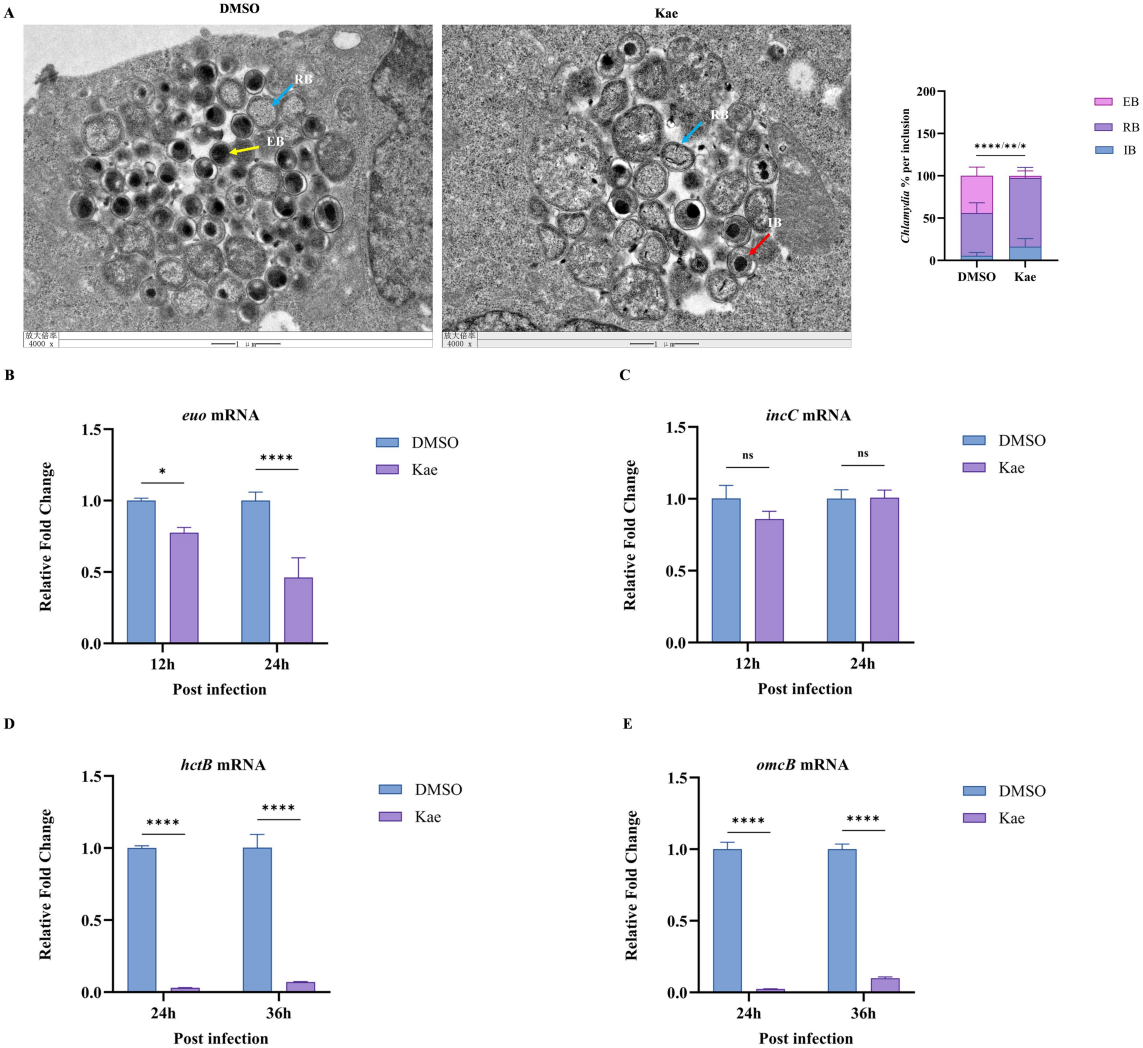
Effects of kaempferol on the developmental cycle of *C. psittaci*. **(A)** HeLa cells were infected with *C. psittaci* at an MOI of 1 and then treated with 40 μM kaempferol or DMSO (control). At 36 h post-infection, samples were processed for transmission electron microscopy (TEM). Representative images show the inclusion morphology, and the relative ratios of elementary bodies (EBs), intermediate bodies (IBs), and reticulate bodies (RBs) were quantified. The experiment was repeated three times with 10 cells counted per group in each repeat. Scale bar = 1 μm. Blue arrows: RBs; red arrows: IBs; yellow arrows: EBs. **(B–E)** HeLa cells were infected with *C. psittaci* at an MOI of 1 and then treated with 40 μM kaempferol or DMSO (control). The relative mRNA expression levels of bacterial genes *euo, incC, hctB,* and *omcB* were quantified by RT-qPCR at 12 h, 24 h, and 36 h post-infection. *p < 0.05, **p < 0.01, ***p < 0.001 vs. control group.

In addition to the EB and RB, there is a transitionary cell form that mediates the transformation between the RB and the EB, the intermediate body (IB) (Chiarelli et al., 2023; Appa et al., 2024). Then, did kaempferol exert on the developmental cycle of *Chlamydia*? To address this, HeLa cells were infected with *C. psittaci* and treated with 40 μM kaempferol and observed by TEM. Compared with the infected control, the kaempferol-treated group showed a significant decrease in the number of EBs and a concomitant increase in intermediate bodies (IBs), which mediate the RB-to-EB transition (Figure 4A). These results indicate that kaempferol suppresses the differentiation of RBs to EBs within the inclusion, leading to an aberrant developmental cycle and reduced the production of new infectious progeny.

Given the facts that kaempferol inhibits the RB-to-EB conversion, and the chlamydial developmental cycle involves key stages, namely EB differentiation, RB proliferation, re-differentiation into EBs, we investigated whether kaempferol affects the transcription of genes regulating these stages. *IncC* forms channels in the inclusion membrane to hijack host nutrients, while *euo* represses late genes (e.g., *omcB*, *hctB*) to ensure sufficient RB replication. *HctB* initiates EB morphological transformation, and *omcB* cross-links outer membrane proteins via disulfide bonds to generate the rigid cell wall of EBs (Shen et al., 2024).

HeLa cells were infected with *C. psittaci* and treated with 40 μM kaempferol, and RT-qPCR was performed to determine the transcription of these genes. Results showed that in the kaempferol-treated group: *euo* transcription was slightly affected in 12 h post-infection but significantly suppressed in 24 h (Figure 4B); *incC* expression was unaffected at these two setting (Figure 4C); and the genes *hctB* and *omcB* responsible for re-differentiation into EBs were markedly downregulated (Figures 4D,E).

Together, these findings demonstrated that kaempferol inhibited intracellular proliferation of *C. psittaci* by blocking the RB-to-EB conversion within the inclusion and downregulating related late-stage (re-differentiation into EBs) genes transcription.

### 3.5 The combination of kaempferol and doxycycline has a synergistic effect

Previous study determined that the minimum inhibitory concentration (MIC) of doxycycline against *C. psittaci* was 0.05–0.25 μg/mL *in vitro* (Butaye et al., 1997). Previous studies have indicated that although some extracts containing kaempferol may not possess significant antibacterial activity on their own, they can potentiate the effects of certain antibiotics (de Freitas et al., 2022). Based on these findings, we designed the following experiment to investigate the inhibitory effects of kaempferol and doxycycline, both individually and in combination, on the intracellular replication of *C. psittaci*. Four experimental groups were established: DMSO control, kaempferol alone, doxycycline alone, and kaempferol combined with doxycycline.

**Figure 5:**
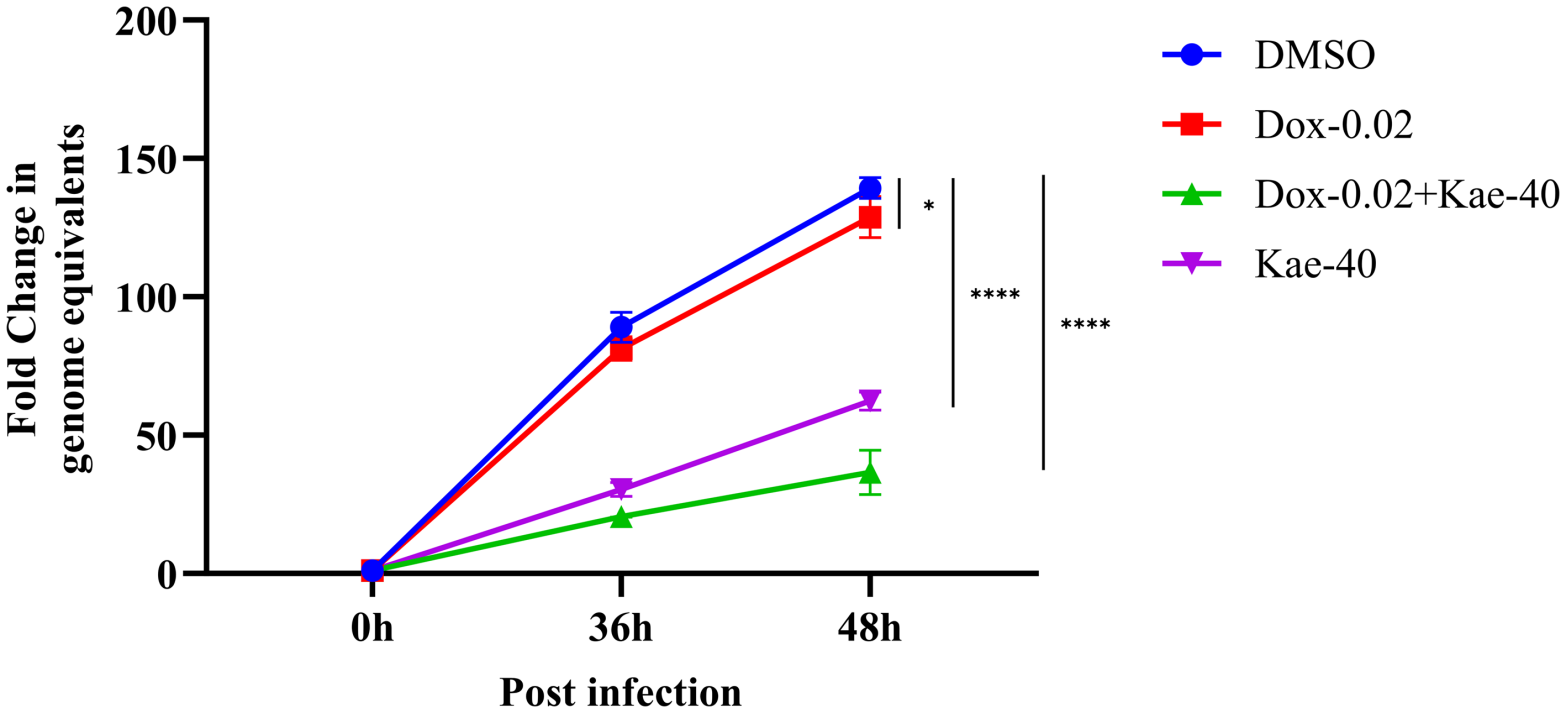
Synergistic effect of kaempferol and doxycycline. HeLa cells were infected with *C. psittaci* at an MOI of 1 for 3 h, and then treated with kaempferol (40 μM), doxycycline (0.02 μM), kaempferol plus doxycycline (40 μM + 0.02 μM), or DMSO (control). The intracellular bacterial yield was assessed by qPCR at the indicated time points (36 h and 48 h). *p < 0.05, **p < 0.01, ***p < 0.001 vs. control group.

The results demonstrated that, compared to the DMSO control group, both kaempferol and doxycycline treatment significantly reduced the infection rate of *C. psittaci* in HeLa cells, indicating that both agents effectively suppress intracellular chlamydial growth at the tested concentrations. Furthermore, when comparing the combination group with each individual treatment group, the reduction in infection rate was markedly greater in the combination group, and this enhanced effect was statistically significant. These findings suggest that kaempferol and doxycycline exert a synergistic inhibitory effect during intracellular *C. psittaci* infection (Figure 5).

## 4 Discussion

As an obligate intracellular pathogen, *Chlamydia* depends on the host cell for essential nutrients, particularly lipids for the synthesis of its own membranes. Studies have shown that *Chlamydia* utilizes type III secretion system (T3SS) effectors (e.g., Inc. proteins) to hijack both vesicular and non-vesicular host lipid trafficking pathways to acquire necessary lipids for growth (Knittler and Sachse, 2015). Key strategies include direct acquisition at membrane contact sites (MCSs) (Elwell and Engel, 2012; Wen et al., 2025), co-opting host lipid transport proteins such as CERT (Elwell et al., 2011; Moldovan et al., 2025), and reorganizing host organelles (e.g., the Golgi apparatus) to create lipid-enriched microenvironments (Elwell et al., 2011). Of particular note, *Chlamydia* likely exploits the host protein CERT-mediated lipid transport pathway to acquire sphingolipids (Elwell et al., 2011). Previous research shows that kaempferol exerts anti-inflammatory effects by inhibiting Src kinase and NF-κB signaling and downregulating lipid metabolism-associated enzymes (e.g., COX-2), supporting its capacity to suppress lipid metabolic pathways (Luo et al., 2012). Our study reveals that kaempferol effectively disrupts the interface between *Chlamydia* and host lipid trafficking, simultaneously impairing recruitment of both lipid droplets and host CERT.

*Chlamydia* requires substantial lipids to construct its outer membrane, inclusion membrane, and to support binary fission. Insufficient lipid supply for instance, due to blockade of host-derived acquisition pathways-compromises proper membrane synthesis, thereby impeding developmental progression, in particular the transition of RBs to EBs and secondary division (Wylie et al., 1997). Yao et al. demonstrated that when *Chlamydia* cannot efficiently utilize host-abundant saturated fatty acids, a shortage of lipid precursors impairs membrane biogenesis and disrupts the developmental cycle (Yao et al., 2015). Lipids serve not only as structural components but also as signaling molecules; lipid deprivation likely directly causes developmental arrest, amplifying inhibitory effects. Disrupting lipid trafficking (e.g., of phosphatidylcholine and sphingolipids)—whether by inhibiting host supply or bacterial lipid-modifying enzymes—specifically blocks RB-to-EB conversion, halting the cycle and drastically reducing infectious EB yield (Strange et al., 2024). Our study shows that kaempferol disrupts the developmental cycle (Figure 4A) causing arrest at the RB stage, likely through obstruction of lipid trafficking.

In addition to blocking lipid trafficking, kaempferol downregulated the transcription of certain genes involving in the RB-to-EB cycle of *C. psittaci.* We examined four stage-specific genetic markers: (i) *incC*, expressed early during infection, encodes the inclusion membrane protein *IncC*, which is likely involved in the initial establishment and formation of the inclusion matrix; (ii) *euo*, expressed from early to mid-stage, encodes the *euo* protein that recognizes and binds specific DNA sequences, repressing represses certain late-gene promoters; (iii) *omcB*, a late-stage gene encoding a structural component of the infectious elementary body (EB), whose protein forms disulfide cross-links to stabilize the outer membrane; (iv) *hctB*, also highly expressed in the late-stage, encoding the histone-like protein *hctB*, which promotes the morphological transition of RBs to EBs (Brickman et al., 1993; Zhang et al., 2000; Rosario and Tan, 2012; Agaisse and Derré, 2014; Hu et al., 2015; Shen et al., 2024). Our study found that kaempferol significantly downregulates the expression of key late developmental genes (*omcB*, *hctB*), leading to a blockade in the RB-to-EB transition. This finding aligns with Schoborg’s “chlamydial persistence” theory, wherein external stress can interrupt the developmental cycle (Schoborg, 2011).

*β*-lactam antibiotics do not directly kill *Chlamydia* but induces a persistent state in which the developmental cycle is arrested: RBs fail to divide and cannot differentiate into elementary bodies (EBs) (Skilton et al., 2009). Similarly, IFN-*γ* triggers host production of indoleamine 2,3-dioxygenase (IDO), depleting tryptophan and stalling chlamydial growth through nutrient limitation (Olivas et al., 2024). Additional studies show that inhibitors such as Bafilomycin A targeting host pathways can effectively suppress inclusion development and markedly reduce the generation of infectious EBs (Ouellette et al., 2011). Together, these observations indicate that developmental arrest represents a common survival strategy adopted by *Chlamydia* under stress, whether triggered by antibiotics, host immune responses, nutrient scarcity, or inhibition of host factors. The dual mechanism of kaempferol, disrupting host lipid trafficking while directly interfering with bacterial development, parallels such strategies, yet exhibits unique attributes that position it as a promising candidate for countering drug-resistant *Chlamydia*. Its ability to impede lipid acquisition mirrors the concept underlying host-directed inhibitors.

This experiment demonstrates that kaempferol not only exhibits intrinsic anti-*Chlamydia psittaci* activity but also significantly enhances the anti-chlamydial efficacy of doxycycline. The synergistic effect observed upon combined use indicates that kaempferol has potential as an adjunctive agent to doxycycline, offering a possible novel combination strategy for the clinical treatment of chlamydial infections. Further studies are warranted to elucidate the molecular mechanisms underlying this synergy and to evaluate its efficacy in additional cell models or *in vivo* systems, thereby facilitating its translational application.

In addition, Kaempferol is a naturally occurring flavonoid widely distributed in edible plants, including tea, broccoli, cabbage, kale, beans, tomato, strawberries, and grapes (Calderón-Montaño et al., 2011). The concentration of kaempferol used in this study (40 μM) was confirmed to be non-cytotoxic, as shown in Figure 2 where cell viability remained above 90% in both HeLa and THP-1 cells. This observation is consistent with the published safety profile of kaempferol. Kashafi et al. reported an IC₅₀ of 10.48 μM in HeLa cells after 72 h treatment, demonstrating its potent anti-cancer activity with a wide therapeutic window (Kashafi et al., 2017). Notably, our study employed a 48 h treatment period, which is shorter than the 72 h exposure used by Kashafi et al. Cytotoxicity is known to be time-dependent-longer exposure times typically result in lower IC₅₀ values as cells accumulate drug-induced damage. Therefore, the higher concentration (40 μM) used in our study remains within the non-cytotoxic range under our experimental conditions. This interpretation is further supported by Karakurt et al., who reported an IC₅₀ of 88.0 μM in HeLa cells (Karakurt et al., 2025), indicating that the cytotoxic threshold of kaempferol in HeLa cells can vary considerably depending on experimental parameters, including treatment duration. Regarding THP-1 macrophages, Huwait et al. explicitly stated that kaempferol is not cytotoxic to these cells (Huwait et al., 2022). These findings support our observation that the 40 μM concentration used in this study is non-cytotoxic to both HeLa and THP-1 cells, while still exerting significant anti-chlamydial activity. The IC₅₀ of kaempferol against *C. psittaci* was not determined. Future studies should address these gaps by conducting comprehensive dose–response analyses to establish both the IC₅₀ in host cells and the MIC against *C. psittaci*, which will provide essential parameters for further preclinical development.

Collectively, Research on natural products such as kaempferol suggests that therapeutic approaches targeting pathogenic mechanisms, rather than merely aiming to kill bacteria, may offer a viable path to alleviating the antibiotic resistance crisis. Our study highlights the considerable potential of kaempferol as a natural-product-based agent against resistant *Chlamydia*, providing a foundation for developing next-generation anti-infectives. Important future directions include using chemoproteomics to identify the direct targets of kaempferol in *Chlamydia* or host cells; validating its efficacy and safety in animal models of chlamydial infection.

## 5 Conclusion

This study demonstrated that the natural flavonoid kaempferol exhibits significant anti-infective activity against *C. psittaci*. Its action is mediated through a dual mechanism: firstly, by interfering with the trafficking of host-derived lipids to the bacterial inclusion, thereby starving the pathogen of essential nutrients; secondly, by disrupting the normal differentiation of RBs into EBs, leading to an aberrant developmental cycle and reduced production of new infectious progeny. These findings not only uncover a novel mechanism of kaempferol’s anti-chlamydial action, but also provide a strong rationale for its development as a promising lead compound for the treatment of *C. psittaci* infections.

## Data Availability

The original contributions presented in the study are included in the article/Supplementary material, further inquiries can be directed to the corresponding authors.

## Footnotes

1
http://tcmspw.com

2
http://www.swisstargetprediction.ch

3
https://www.genecards.org/

4
https://www.omim.org/

5
https://bioinfogp.cnb.csic.es/tools/venny/index.html

6
https://cn.string-db.org/

## Supplementary material

FIGURE S1 Identification of common targets between active drug 773 components and psittacosis, and their protein-protein interaction network. **(A)** 774 Venn diagram showing the overlap between targets of active drug components and 775 psittacosis-related targets. **(B)** Protein-protein interaction (PPI) network of common 776 targets; node size and color intensity correspond to degree values, with larger and 777 darker nodes indicating stronger associations within the network.

FIGURE S2 Cytotoxicity of quercetin and luteolin in HeLa and 780 THP-1 cells. **(A, B)** The viability of HeLa and THP-1 cells was measured by a CCK-8 781 assay after 24 h and 48 h of treatment with different concentrations of quercetin (16, 782 32, 64 μM) or luteolin (10, 20, 40 μM), with DMSO-treated cells serving as the 783 control. ****p* < 0.001, *****p* < 0.0001 vs control group.
