# Correction: Kaempferol inhibits *Chlamydia psittaci* proliferation by blocking lipid transport and RB-EB differentiation

**DOI:** 10.3389/fmicb.2026.1853740

**Published:** 2026-04-28

**Authors:** Frontiers Production Office

**Affiliations:** Frontiers Media SA, Lausanne, Switzerland

**Keywords:** *Chlamydia psittaci*, developmental cycle, kaempferol, lipid droplets, RB-EB differentiation

Affiliation “RGUHS, India” was erroneously included for reviewer Roshan Pais. The correct affiliation is “CSI Lombard Memorial Hospital, Udupi, Rajiv Gandhi University of Health Sciences, India.”

The original version of this article has been updated.

