# Supplementary material for: Kaempferol inhibits Chlamydia psittaci proliferation by blocking lipid transport and RB-EB differentiation
Source: Front Microbiol. 2026 Mar 13;17:1783916. doi: 10.3389/fmicb.2026.1783916 (PMC13021591; doi:10.3389/fmicb.2026.1783916)
Supplement: Supplementary file 1 [file Data_Sheet_1.PDF]

Table S1 List of active ingredients of the drug

| Ingredient No. | Ingredient |
|---|---|
| 1 | wogonin |
| 2 | (2R,3R,4S)-4-(4-hydroxy-3-methoxy-phenyl)-7-methoxy-2,3-dimethylol-tetralin-6-ol |
| 3 | (3R,4R)-3,4-bis[(3,4-dimethoxyphenyl)methyl]oxolan-2-one |
| 4 | (+)-pinoresinol monomethyl ether |
| 5 | ACon1_001697 |
| 6 | (+)-pinoresinol monomethyl ether-4-D-beta-glucoside_qt |
| 7 | 3beta-Acetyl-20,25-epoxydammarane-24alpha-ol |
| 8 | Mairin |
| 9 | FORSYTHINOL |
| 10 | (-)-Phillygenin |
| 11 | hyperforin |
| 12 | Onjixanthone I |
| 13 | beta-sitosterol |
| 14 | kaempferol |
| 15 | arctiin |
| 16 | luteolin |
| 17 | bicuculline |
| 18 | quercetin |
| 19 | acacetin |
| 20 | (2R)-7-hydroxy-5-methoxy-2-phenylchroman-4-one |
| 21 | baicalein |
| 22 | 5,7,2,5-tetrahydroxy-8,6-dimethoxyflavone |
| 23 | Carthamidin |
| 24 | Dihydrobaicalin_qt |
| 25 | Eriodyctiol (flavanone) |
| 26 | Salvigenin |
| 27 | 5,2',6'-Trihydroxy-7,8-dimethoxyflavone |
| 28 | 5,7,2',6'-Tetrahydroxyflavone |
| 29 | Skullcapflavone II |
| 30 | oroxylin a |
| 31 | Panicolin |

| | |
|---|---|
| 32 | 5,7,4'-Trihydroxy-8-methoxyflavone |
| 33 | NEOBAICALEIN |
| 34 | DIHYDROOROXYLIN |
| 35 | Moslosooflavone |
| 36 | 11,13-Eicosadienoic acid, methyl ester |
| 37 | 5,7,4'-trihydroxy-6-methoxyflavanone |
| 38 | 5,7,4'-trihydroxy-8-methoxyflavanone |
| 39 | rivularin |
| 40 | estrone |
| 41 | 11,14-eicosadienoic acid |
| 42 | sitosterol |
| 43 | Stigmasterol |
| 44 | gondoic acid |
| 45 | CLR |
| 46 | (+)-catechin |
| 47 | Glycyrol |
| 48 | Spinasterol |
| 49 | Licochalcone B |
| 50 | liquiritin |
| 51 | Glabridin |
| 52 | Phaseol |
| 53 | Machiline |
| 54 | l-SPD |
| 55 | leucopelargonidin |
| 56 | Herbacetin |
| 57 | Resivit |
| 58 | delphinidin |
| 59 | Mandenol |
| 60 | 24-Ethylcholest-4-en-3-one |
| 61 | poriferast-5-en-3beta-ol |
| 62 | Diosmetin |
| 63 | naringenin |
| 64 | taxifolin |
| 65 | campest-5-en-3beta-ol |

| | |
|---|---|
| 66 | eriodictyol |
| 67 | Genkwanin |
| 68 | Pectolinarigenin |
| 69 | (+)-Leucocyanidin |
| 70 | Truflex OBP |
