# Supplementary figures and images for: Kaempferol inhibits Chlamydia psittaci proliferation by blocking lipid transport and RB-EB differentiation
Source: Front Microbiol. 2026 Mar 13;17:1783916. doi: 10.3389/fmicb.2026.1783916 (PMC13021591; doi:10.3389/fmicb.2026.1783916)

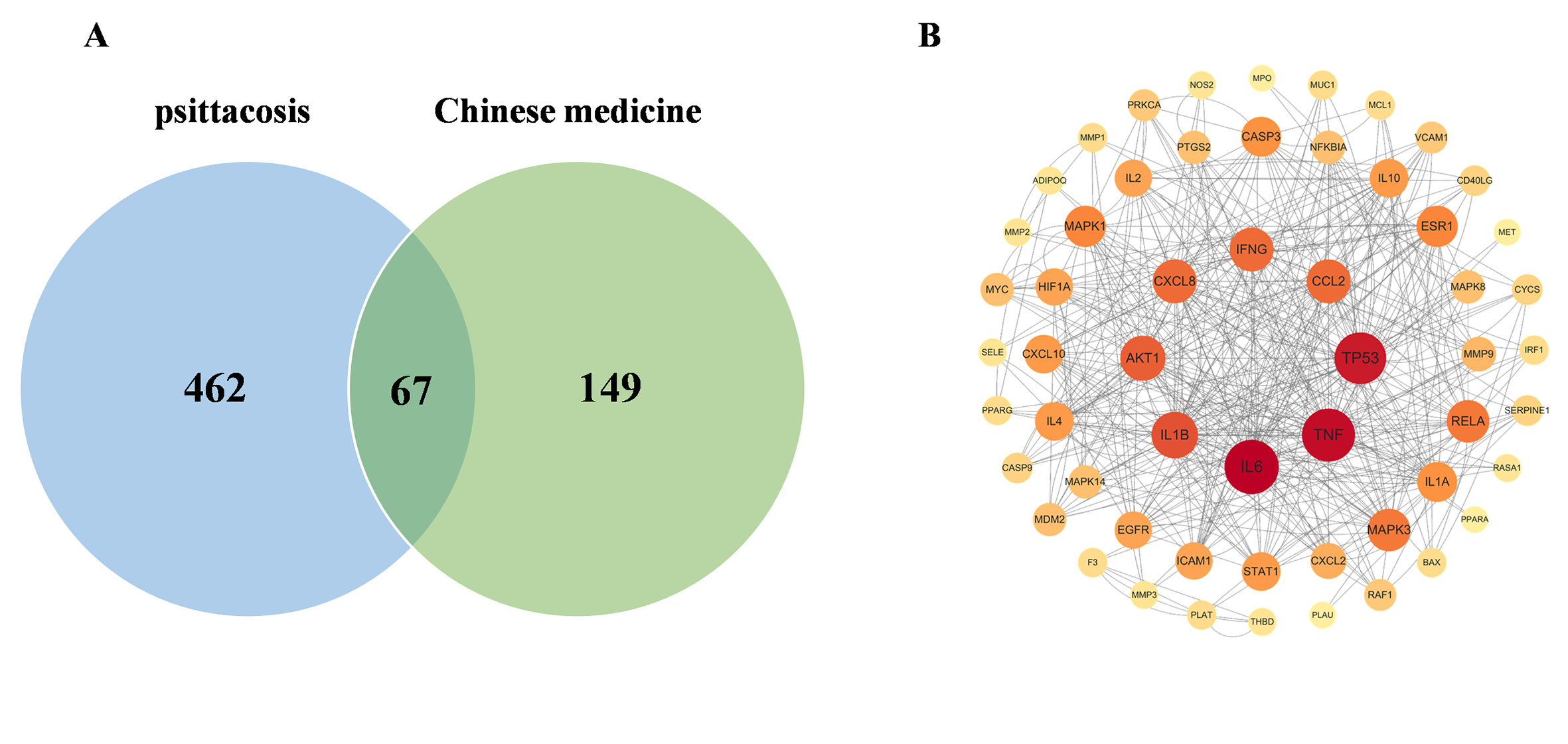

Supplement: FIGURE S1 — Identification of common targets between active drug 773 components and psittacosis, and their protein-protein interaction network. (A) 774 Venn diagram showing the overlap between targets of active drug components and 775 psittacosis-related targets. (B) Protein-protein interaction (PPI) network of common 776 targets; node size and color intensity correspond to degree values, with larger and 777 darker nodes indicating stronger associations within the network. [file Image_1.TIF]

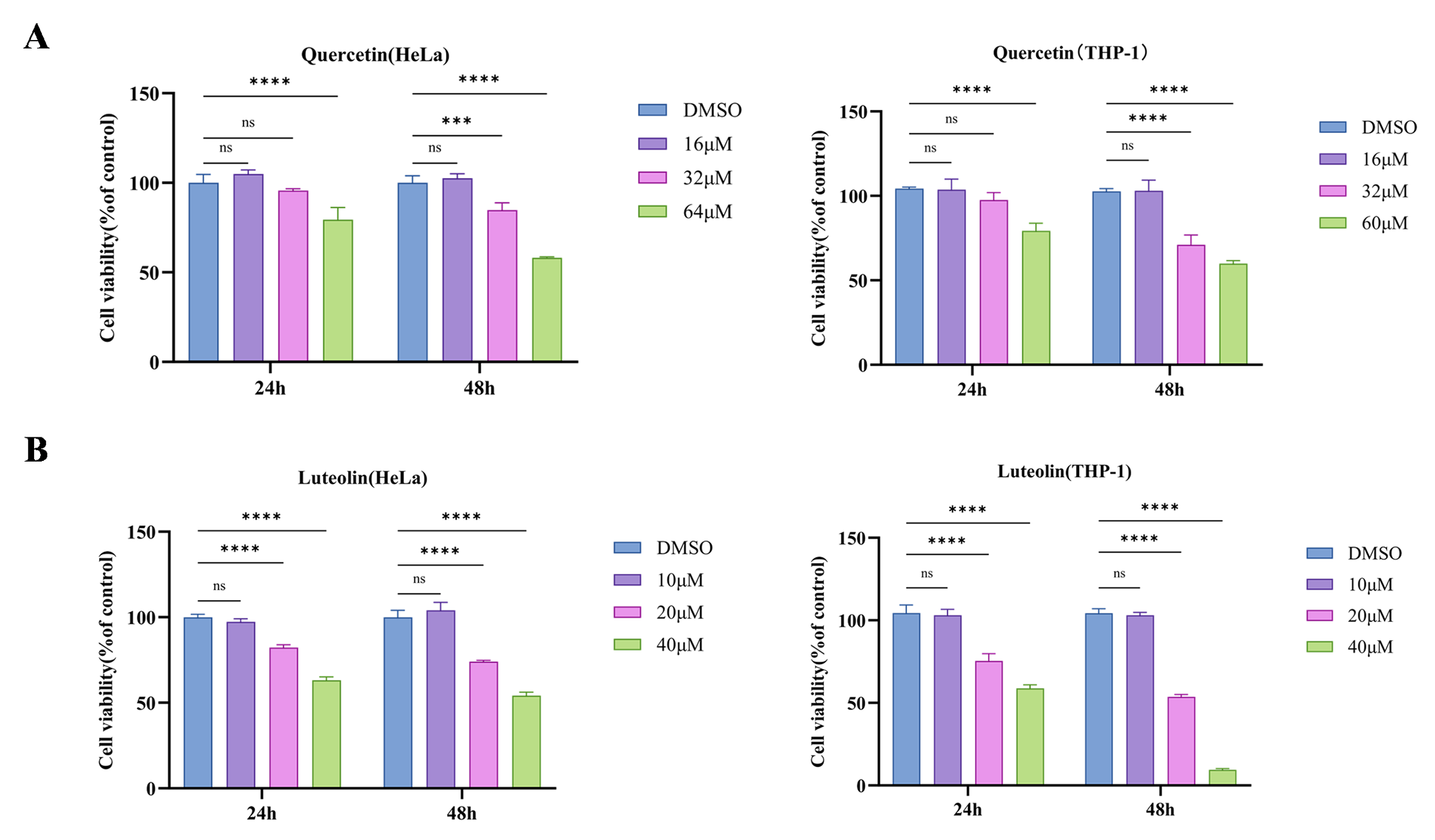

Supplement: FIGURE S2 — Cytotoxicity of quercetin and luteolin in HeLa and 780 THP-1 cells. (A, B) The viability of HeLa and THP-1 cells was measured by a CCK-8 781 assay after 24 h and 48 h of treatment with different concentrations of quercetin (16, 782 32, 64 μM) or luteolin (10, 20, 40 μM), with DMSO-treated cells serving as the 783 control. ***p < 0.001, ****p < 0.0001 vs control group. [file Image_2.TIF]
